# Nonlocal Effects and Slip Heat Flow in Nanolayers

**DOI:** 10.1038/s41598-017-10416-9

**Published:** 2017-08-29

**Authors:** Chuan-Yong Zhu, Wei You, Zeng-Yao Li

**Affiliations:** Key Laboratory of Thermo-Fluid Science and Engineering of Ministry of Education, School of Energy and Power Engineering, Xi’an Jiaotong University, Xi’an, 710049 P.R. China

## Abstract

Guyer-Krumhansl (G-K) equation is a promising macroscopic model to explore heat transport in nanoscale. In the present work, a new nonlocal characteristic length is proposed by considering the effects of heat carriers-boundaries interactions to modify the nonlocal term in G-K equation, and a slip heat flux boundary condition is developed based on the local mean free path of heat carriers. Then an analytical solution for heat flux across 2-D nanolayers and an in-plane thermal conductivity model are obtained based on the modified G-K equation and the slip heat flux boundary. The predictions of the present work are in good agreement with our numerical results of direct simulation Monte Carlo (DSMC) for argon gas nanolayer and the available experimental data for silicon thin layers. The results of this work may provide theoretical support for actual applications of G-K equation in predicting the thermal transport properties of nanolayers.

## Introduction

Nanoscale systems with dimensions comparable to the heat carrier mean-free path has drawn tremendous attention in last two decades due to their unique properties and potential for a wide variety of applications in thermoelectric devices^1, 2^, electronic devices^3^ and micro-sensors^4^. For example, the phonon transport in a silicon layer is significantly suppressed by the attenuating thickness resulting in a decrease in the in-plane thermal conductivity^5–9^, which makes it a promising candidate for enhancing the figure of merit when applied to thermoelectric devices. In addition, unique thermal properties are also observed in suspended grapheme layers and other two-dimensional thin layers^10–12^. Thus, analysis of the heat transport in two-dimensional thin layers has become a topic of current interest.

For heat transport in the domain with the dimension comparable or even smaller than the mean-free path of heat carriers, interactions between heat carriers and boundaries become more pronounced, and experiments have demonstrated that the classical Fourier law is invalid for heat transport in this regime^13^. In order to tackle the related thermal phenomena in nanosystems, such as nonlocal effect, fruitful efforts have been made to develop substitutes in theory on mirco- and nanoscale heat transport at microscopic, mesoscopic and macroscopic level. Even though the methods at microscopic and mesoscopic level, such as the first-principle calculations, molecular dynamics simulations and phonon Boltzmann equation, are able to explain some thermal phenomena at nanoscale, however these methods are computationally expensive. In recent years, some researchers have focused their attention on developing macroscopic methods which can describe the heat transport at nanoscale. There are several constitutive equations at macroscopic level for nanoscale heat transfer in literature, including the phonon hydrodynamics model^14, 15^, the thermomass model^16^ and the dual-lag model^17^. It is worth noticing that all these models can be derived by starting from the Boltzmann transport equation^18^ or purely on macroscopic grounds^19^. Among these macroscopic models, the phonon hydrodynamic model is the most natural and direct production from the Boltzmann equation, avoiding empirical parameters or pure mathematical terms in other models^20^. In this work, we will focus our attention on the phonon hydrodynamic model.

The phonon hydrodynamic model was first derived by Guyer and Krumhansl (G-K) from the kinetic theory of phonons^14, 21^ to describe the phonon heat transport in dielectric crystals at low temperatures. In last few years, numerous subsequent theoretical studies have been done to refine and extend the original phonon hydrodynamic model (G-K equation), and a general G-K like equation with gradient terms of heat flux to account for the nonlocal effect in nanoscale is proposed^15, 22^ as shown in Eq. (1). In fact, an equation of this form can also be derived from extended irreversible thermodynamics^23^ and other generalized thermodynamic formalisms^24^.

1 $$\tau \frac{\partial {\boldsymbol{q}}}{\partial t}+{\boldsymbol{q}}=-{\lambda }_{\infty }\nabla T+{l}_{GK}^{2}({\nabla }^{2}{\boldsymbol{q}}+2\nabla \nabla \cdot {\boldsymbol{q}}),$$

where *τ* is the relaxation time, ***q*** is the local heat flux, *T* is the temperature, *λ*_∞_ is the bulk thermal conductivity and *l*_*GK*_ represents the characteristic length of nonlocal effect (a phenomenological coefficient) which is related to the mean-free path of heat carriers^18^. Equation (1) is slightly different from the original phonon hydrodynamic model, which is a phenomenological formulation of phonon hydrodynamics by avoiding the complexities of a detailed microscopic description. The simplification makes Eq. (1) more practical for modeling the heat transport in nanosystems with a reasonable physical description. For convenience, we still use the name of G-K equation for Eq. (1).

**Figure 1:**
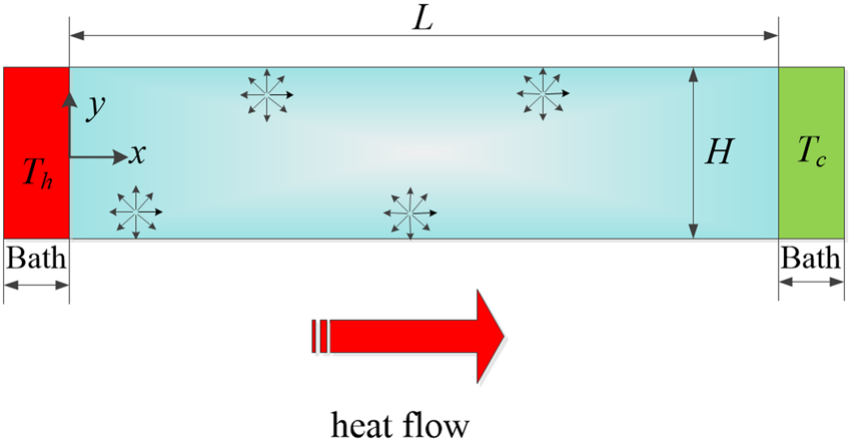
Sketch of a two-dimensional nanolayers. *H* and *L* are the thickness and length of the system, where *L* ≫ *H*. The arrow stands for the direction of the local heat flux along the x-axis.

Recently, based on Eq. (1), heat conduction in nanolayers and nanowires has been extensively investigated^25–28^. It is revealed that the heat-flux profile in nanolayers may exhibit a parabolic profile as the velocity distribution of rarefied gas flows or microscale flows in microchannels with the rarefied effect^20^.

2 $${{\bf{v}}}_{w}=c^{\prime} l\frac{\partial {{\bf{v}}}_{b}}{\partial n}$$

where **v**_*b*_ represents the fluid speed in the bulk of the micro-channel, **v**_*w*_ is the velocity of the fluid on the walls, *l* is the mean free path of the fluid particles, *n* denotes the unit normal direction of the wall and, *c*′ the slip coefficient determined by the surface properties of the walls. Analogous to Eq. (2), a heat flux slip boundary condition in nanolayers was developed^26–28^.

3 $${{\boldsymbol{q}}}_{w}=C{l}_{GK}{(\frac{\partial {\boldsymbol{q}}}{\partial y})}_{y=0.5H},$$

where *C* is a non-dimensional coefficient describing specular and diffusive reflections of heat carriers, *H* is the length scale (nanolayer thickness, see Fig. 1). Meanwhile, a parallel effort^29^ has also been made to prove the legitimacy of the slip boundary condition of heat flux on the surface by deriving from the discrete Boltzmann transport equation.

Extensive explorations on heat transport in nanofilms^22^, nanowires^27^ and porous nanostructures^30^ based on Eq. (1) indicates that the G-K equation is a promising tool in modeling thermal transport properties of nanostructures. However, Eq. (1) is proposed by a phenomenological way with the characteristic length of nonlocal effect *l*_*GK*_ as a purely phenomenological parameter whose microscopic features are not yet fully understood. In order to keep the maximum simplicity in the phenomenological description of the phonon hydrodynamics model, *l*_*GK*_ in Eqs (1) and (3) was regarded as the average mean-free path for bulk material, i.e., *l*_∞_ in many studies^15, 22, 25^. However, Carlomagno *et al*.^28^ suggested that *l*_*GK*_ may have an order of magnitude which differs from *l*_∞_. Up to now, little work has reported the magnitude of *l*_*GK*_ and the dominating factors which may influence it. In addition, few studies have been conducted to investigate the heat flux slip boundary and heat flux profiles quantitatively by experiments or numerical simulations.

The goals of this paper are to look deep into the physical mechanism of *l*_*GK*_ from a microscopic point of view, and to propose theoretical prediction models which may be useful in practical applications.

This paper is organized as follows. In section 2, we will propose a new characteristic length *l*_*GK*_ by considering the boundary scattering of heat carriers, and then derive an analytical solution for heat flux profile and an in-plane thermal conductivity model of nanolayers. In section 3, we will compare the proposed heat flux profile and the thermal conductivity model with DSMC results of argon gas layers and the available experimental data of silicon thin layers. In addition, some existing models presented in previous literature will be discussed. And then, we will draw our conclusions in section 4. Finally, the numerical method is introduced in section 5.

## Theoretical Models

In the present section, we use Eq. (1) to study the heat transport in a 2-D nanolayer as shown in Fig. 1, in which the size along *x*-axis direction is much larger than the mean-free path of heat carriers.

Equation (1) will reduce to the following form under the steady-state condition without internal heat source.

4 $${\boldsymbol{q}}=-{\lambda }_{\infty }\nabla T+{l}_{GK}^{2}{\nabla }^{2}{\boldsymbol{q}}.$$

It is worth noticing that the nonlocal term in Eqs (1) and (4) is caused by the size effect since Eq. (4) reduces to the Fourier law when *l*_*GK*_ vanishes. So the value of *l*_*GK*_ should be dependent on the size of the nanosystems. Tzou^17^ suggested that the nonlocal effect is confined within a physical domain close to the boundary, where the nonlocal response is more pronounced because of the active interactions between the heat carriers and the boundary. According to the above analysis, the nonlocal characteristic length *l*_*GK*_ should be dependent on the size of nanosystems and effect of the interactions between the heat carriers and the boundary. Furthermore, in the original phonon hydrodynamics model, the coefficient ${l}_{GK}^{2}$ has a dimension of length squared, and could be expressed as ${l}_{GK}^{2}={v}^{2}\tau {\tau }_{1}$ ^31^, where *v* is the heat carriers speed, *τ*_1_ is the relaxation time related to collisions between heat carriers and boundary, and *τ* is the relaxation time of interactions between heat carriers. So, in this work, we discard the hypothesis of ${l}_{G{\rm{K}}}=\sqrt{{l}_{\infty }{l}_{\infty }}={l}_{\infty }$ and define the nonlocal characteristic length as Eq. (5) by considering the heat carriers-boundary collisions.

5 $${l}_{GK}=\sqrt{{l}_{w}{l}_{\infty }},$$

where *l*_*w*_ is the local mean-free path of heat carriers adjacent to the wall (*y* = ±0.5*H*), which is dominated by the collisions between heat carriers and the boundary (depending on the size of the system).

In bulk materials, the probability of a heat carrier travelling a distance of *x* between successive collisions can be expressed as refs 32–34

6 $$P(x,x+{\rm{\Delta }}x)=\exp (-\frac{x}{{l}_{\infty }}){\rm{\Delta }}\frac{x}{{l}_{\infty }}$$

The value of the unconfined, conventional mean-free path can be obtained by integrating Eq. (6) with respect to *x* from zero to infinity. As a wall is included in the system, however, some heat carriers will hit the wall and their flight paths will be terminated by the wall, and the mean free path of all the heat carriers are smaller than *l*_∞_. For practical nanowires and nanolayers, the effective mean free path needs to be integrated over the sphere angle. In a 2-D nanolayer as shown in Fig. 1, the local mean free path of heat carriers can be expressed as refs 32–34

7 $$\frac{{l}_{y}}{{l}_{\infty }}=1+\frac{1}{2}[(\alpha -1)\exp (-\alpha )+(\beta -1)\exp (-\beta )-{\alpha }^{2}{E}_{i}(\alpha )+{\beta }^{2}{E}_{i}(\beta )],$$

where

8 $$\begin{array}{ccc}\alpha =\frac{H/2-y}{{l}_{\infty }}, & \beta =\frac{H/2+y}{{l}_{\infty }}, & {E}_{i}(z)={\int }_{1}^{\infty }{t}^{-1}\exp (-tz)dt\end{array}.$$

The value of *l*_*w*_ in a 2-D thin layer can be obtained from Eqs (7) and (8) at *y* = ±0.5*H*.

As in ref. 25, an assumption that the temperature gradient (*dT*/*dx*) is constant and the heat flux only flows along the *x*-axis is made in this paper as well. To make the problem mathematically tractable, we define the following non-dimensional variables with *q*_0_ = −*λ*_∞_ *dT*/*dx* and *H* as

9 $${q}^{\ast }=q/{q}_{0},{y}^{\ast }=y/H,Kn={l}_{\infty }/H,$$

where *Kn* designates the Knudsen number. Substituting Eq. (9) into Eq. (4) yields

10 $${C}_{0}^{2}K{n}^{2}\frac{\partial {q}^{\ast }}{\partial {y}^{\ast 2}}-{q}^{\ast }=-1,$$

where *C*_0_ = *l*_*GK*_/*l*_∞_. To solve Eq. (10), the symmetric condition at the center of the nanolayer and the slip heat flux boundary conduction on the solid surface, are employed.

11 $$\begin{array}{c}{\frac{\partial {q}^{\ast }}{\partial {y}^{\ast }}|}_{{y}^{\ast }=0}=0\\ {{q}^{\ast }|}_{{y}^{\ast }=\pm 0.5}={q}_{w}^{\ast }\end{array},$$

where ${q}_{w}^{\ast }$ is the heat flux on the solid surface. The heat flux profiles in the in-plane thin layers can be obtained analytically by solving Eq. (10), once ${q}_{w}^{\ast }$ is given. With a fixed temperature gradient, heat flux is proportional to thermal conductivity which is proportional to the heat carrier mean-free path. Therefore, we assume

12 $${q}_{w}^{\ast }=\frac{{q}_{w}}{{q}_{0}}=\frac{{\lambda }_{w}}{{\lambda }_{\infty }}=\frac{{l}_{w}}{{l}_{\infty }}.$$

Then, coupling with Eqs (11) and (12), Eq. (10) can be solved analytically and the solution can be written as

13 $${q}^{\ast }({y}^{\ast })=1+({q}_{w}^{\ast }-1)\frac{\cosh ({y}^{\ast }/(Kn\sqrt{{q}_{w}^{\ast }}))}{\cosh (0.5/(Kn\sqrt{{q}_{w}^{\ast }}))}.$$

With the non-dimensional heat flux distribution obtained by Eq. (13), the total heat flux along nanolayer can be defined as

14 $${Q}_{tot}^{\ast }=2{\int }_{0}^{0.5}{q}^{\ast }({y}^{\ast })d{y}^{\ast }.$$

And then we can calculate the dimensionless in-plane thermal conductivity of the nanolayer as

15 $$\frac{{\lambda }_{eff}}{{\lambda }_{\infty }}=1+2\sqrt{{q}_{w}^{\ast }}({q}_{w}^{\ast }-1)Kn\,\tanh (0.5/(Kn\sqrt{{q}_{w}^{\ast }})).$$

As an alternative way, the heat flux distribution and the in-plane thermal conductivity in nanolayer were obtained in the previous study^27^ by using Eq. (3) with *l*_*GK*_ = *l*_∞_ instead of Eq. (12) as the slip heat flux boundary condition, which can be expressed as

16 $${q}^{\ast }({y}^{\ast })=1-[\frac{1}{1+C\,\tanh (0.5/Kn)}]\frac{\cosh ({y}^{\ast }/Kn)}{\cosh (0.5/Kn)},$$

17 $$\frac{{\lambda }_{eff}(Kn)}{{\lambda }_{\infty }}=1-\frac{2Kn\,\tanh (0.5/Kn)}{1+C\,\tanh (0.5/Kn)},$$

where *C* stands for the non-dimensional coefficient (accounting for specular and diffusive reflections) as in Eq. (3), which can be defined as ref. 28

18 $$C=2(\frac{1+P}{1-P}),$$

where *P* represents the fraction of heat carriers reflected back with a specular reflection from a solid surface. Since the value of *P* is between 0 and 1, *C* in Eq. (18) varies from 2 to ∞. If *P* = 0.0 (*C* = 2), the incident heat carriers are reflected diffusely. If *P* = 1.0 (*C* → ∞) the reflection is perfectly specular. It is observed that when *C* → ∞, Eq. (16) turns to be ${q}_{w}^{\ast }=1$ and Eq. (17) to be *λ*_*eff*_(*Kn*)/*λ*_∞_ = 1 which means that perfectly specular boundary scatterings (*C* → ∞) have no influence on the heat transport in 2-D thin layers. In addition, as a special case, if *C* = 0 is adopted, according to Eq. (3) the heat flux on the solid surface vanishes (***q***_*w*_ = 0), Eq. (16) and Eq. (17) turn into the non-slip model^28^.

## Results and Discussion

As mentioned in section 1, Eq. (1) as a generalized heat conduction equation can be derived from the BTE under appropriate assumptions or purely on macroscopic grounds without making explicit reference to physical nature of heat carriers^20, 23, 35^. In this section, we will validate the proposed heat flux profile and the in-plane thermal conductivity model quantitatively with DSMC results of argon gas layers and the available experimental data of silicon thin layers.

At first, a DSMC study on heat conduction of argon gas confined in a long channel as shown in Fig. 1 is conducted. In this model, the left and the right sides are kept at constant temperatures *T*_*h*_ and *T*_*c*_, respectively, and the top and the bottom walls are adiabatic with diffuse reflection.

**Figure 2:**
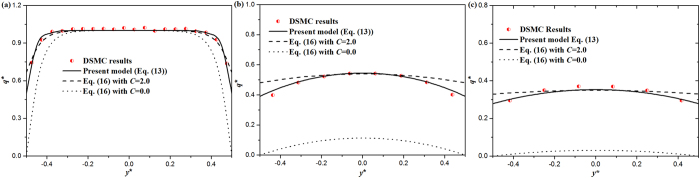
Compasrions of the distribution of heat flux in argon nanolayers obatined by different models, i.e., the present model Eq. (13) (solid line), the previous model Eq. (16) with *C* = 2 (dash line), the previous model Eq. (16) with *C* = 0 (dot line), and DSMC results (scatters) with various *Kn* at 300 K: (**a**) *Kn* = 0.05. (**b**) *Kn* = 1.0. (**c**) *Kn* = 2.0.

Figure 2 shows the non-dimensional heat flux distributions across thin gas layers predicted by different models and DSMC results with various *Kn* at 300 K. Apparently, the influence of boundary scattering on heat transport in nanolayer is pronounced, especially for nanolayers at large *Kn*. When *Kn* is small (for example *Kn* = 0.05), there is a steep reduction in heat flux near solid surfaces, while the heat flux in the center of the layer has no different with that obtained from Fourier law. This is because, when *Kn* is small, the effect of the interactions between heat carriers and boundary is confined within a physical domain adjacent to the walls. With the increase of *Kn*, the heat flux in the center of the layer decreases dramatically, since the Knudsen layer pervades the main part of the cross section. For *Kn* = 2.0 (see Fig. 2(c)), there is about 65% reduction in the heat flux at the center of the layer compared with the value based on the Fourier law. Figure 2 also illustrates that the heat flux profiles predicted by the present model are in excellent agreement with the DSMC results, which indicates that the proposed nonlocal characteristic length ${l}_{GK}=\sqrt{{l}_{GK}{l}_{\infty }}$ and the slip heat flux boundary condition shown in Eq. (12) can capture the behavior of heat transport in 2-D thin layers accurately.

The discrepancy between Eqs (13) and (16) is due to the different way that the boundary conditions (namely, in the form of Eq. (3) for the Eq. (16), and Eq. (12) for Eq. (13)), and the different nonlocal characteristic lengths (namely, *l*_*GK*_ = *l*_∞_ for Eq. (16), and ${l}_{GK}=\sqrt{{l}_{GK}{l}_{\infty }}$ for Eq. (13)) have been used. Note that when *y* = ±0.5*H* the heat flux on the wall is only determined by the slip heat flux boundary. It is observed that Eq. (16) with *C* = 2 overestimates the heat flux near the surface, which means that as an analogous formula, Eq. (3) could not describe the heat flux near the surface properly. Furthermore, according to Eqs (13) and (16), the heat flux in the center of the layers depends on both the slip heat flux boundary and the nonlocal characteristic length. From Fig. 2, it is seen that even with the inaccurate slip heat flux boundary (namely, Eq. (3)), Eq. (16) with *C* = 2 could well predict the heat flux near the center of the gas layers in a wide range of *Kn*. Therefore, it is logically concluded that this coincidence in value is caused by the assumption of *l*_*GK*_ = *l*_∞_.

From Fig. 2(a–c), Eq. (16) with *C* = 0 seriously underestimates the heat fluxes, which implies that assuming the heat flux vanishing on the wall will cause considerable error. This is logical because as expressed in Eq. (13), the mean free path of carriers near the walls is nonzero, i.e. the heat flux on the walls is nonzero. So, the non-slip heat flux boundary condition is unreasonable when exploring the heat transport properties of nanostructures by using the G-K like equation.

**Figure 3:**
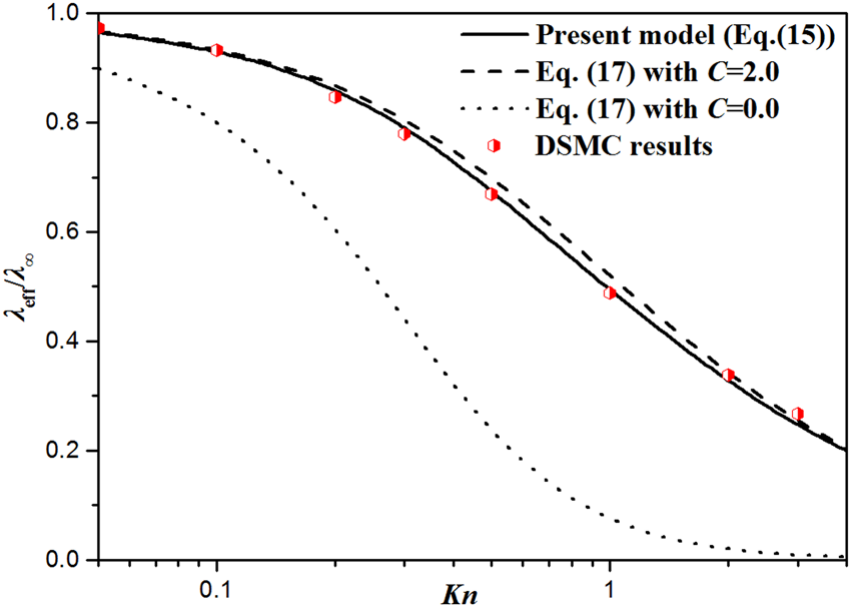
Dimensionless in-plane thermal conductivity of argon gas layers obatined by different models, i.e., the present model Eq. (15) (solid line), the previous model Eq. (17) with *C* = 2 (dash line), the previous model Eq. (17) with *C* = 0 (dot line), and DSMC results (scatters) with various *Kn* at 300 K.

In Fig. 3, the in-plane thermal conductivity of thin gas layers predicted by different models and the DSMC results are plotted with various *Kn*. It is illustrated that the present thermal conductivity model of Eq. (15) exhibits the highest accuracy among the three models, while Eq. (17) with *C* = 0.0 severely underestimates the in-plane thermal conductivity. Though Eq. (16) with *C* = 2.0 could not catch the trend of heat flux near wall accurately, Eq. (17) with *C* = 2.0 can predict the in-plane thermal conductivity of 2-D thin layers quantitatively, with slight deviation in a wide range of *Kn*.

**Figure 4:**
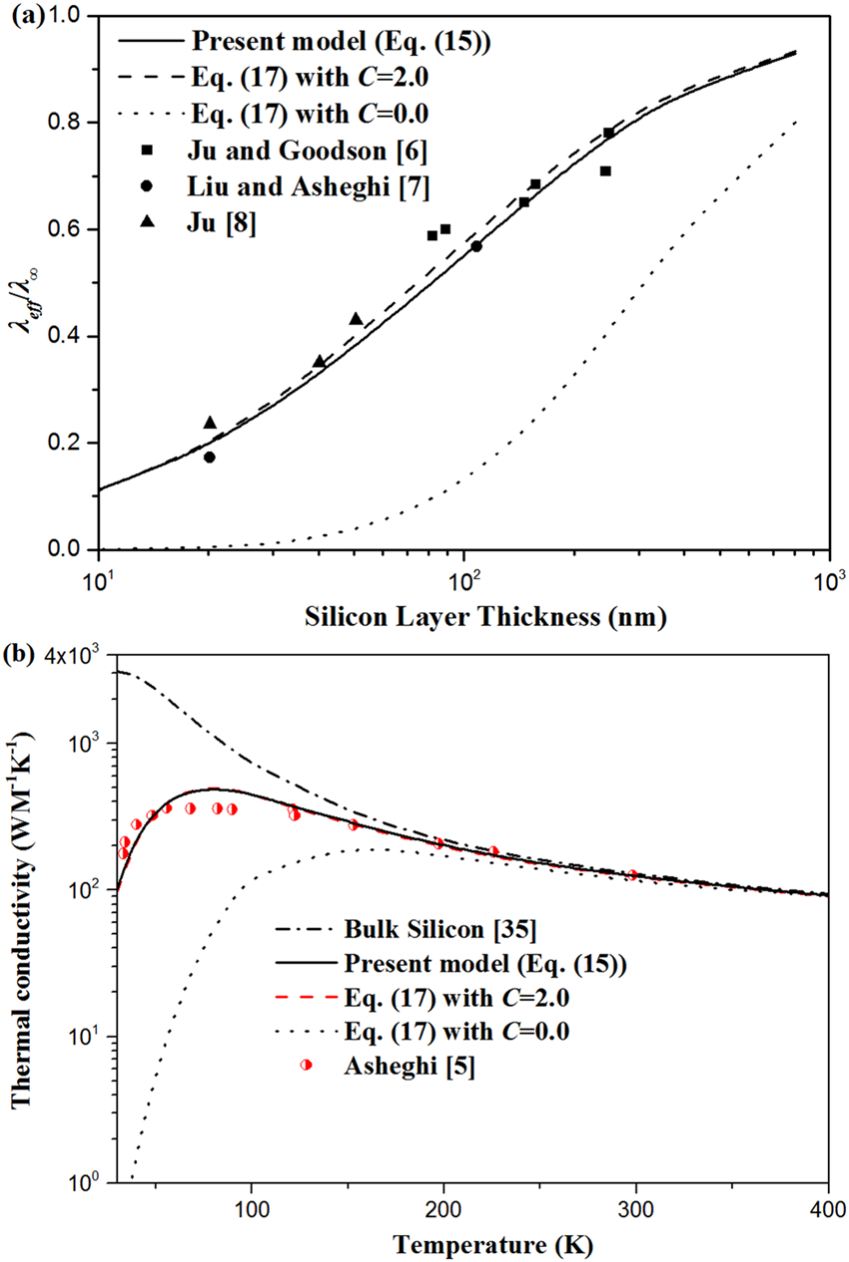
Dimensionless in-plane thermal conductivity of silicon thin layers: (**a**) Comparison of the in-plane thermal conductivity of silicon layers obtained by different models (lines, same as Fig. 3), and experimental data from Ju and Goodson (squares), Liu and Asheghi (dots), and Ju (triangles) versus thickness at 300 K. (**b**) Comparison of the in-plane thermal conductivity versus temperature with thickness of 1.6 μm obtained by different models (lines same as Fig. 3) with available experimental data from Asheghi (scatters), and bulk thermal conductivity of silicon from Sellitto, A. (dash dot line).

The comparisons of different in-plane thermal conductivity models with the available experimental data for silicon nanolayers^5–8^ are presented in Fig. 4. It is also assumed that the phonon scattering on the boundary is diffuse at temperature higher than 30 K^5^. In fact, the phonon mean-free path depends both on the phonon frequency and on the kind of collisions. In such a way, several different relevant averages may be used for it. In this study, for predicting the thermal conductivity of silicon nanolayers, the values of phonon mean-free path are obtained from the relation ${\lambda }_{\infty }=1/3{c}_{v}v{l}_{\infty }$, where *λ*_∞_, *c*_*v*_, and *v* are experimental values from ref. 35. As shown in Fig. 4, the present model Eqs (15) and (17) with *C* = 2.0 are also in agreement with experimental data of silicon nanolayers with various thickness at 300 K and those of silicon layer with thickness of 1.6 μm with the temperature ranging from 30 to 300 K.

## Conclusions

In this paper, we have studied the in-plane heat transport in 2-D long nanolayers based on the phenomenological phonon hydrodynamics approach, i.e., G-K equation. A new nonlocal characteristic length, *l*_*GK*_, is defined by considering the interactions between heat carriers and boundaries. Based the modified G-K equation, a heat flux model and an in-plane thermal conductivity model for nanolayers are proposed. Validation calculations show that the effect of interactions between heat carriers and boundaries should be considered both in a proper boundary condition and in the nonlocal characteristic length to capture the trend of heat flux near the walls more accurately using G-K equation. As an alternative model, Eq. (17) with *C* = 2.0 model can be used to predict the in-plane thermal conductivity of 2-D nanolayers quantitatively, even though Eq. (16) with *C* = 2.0 leads to some deviations when applied to predict the heat flux profile.

## Method

In this work, the numerical results of heat flux and the in-plane thermal conductivity of argon gas layers are calculated by the DSMC method. The DSMC method proposed by Bird^36^ is one of the most successful methods for describing the rarefied gas flow and heat transfer. In this method, each simulated molecule represents substantial real molecules to reduce the number of simulated molecule and then the computational effort. The main idea and details of the DSMC can be found in ref. 36. In this work, the initial pressure and temperature of the gas confined in a long channel as shown in Fig. 1, are *p* = 1.01 × 10^5^ Pa and *T*_*ref*_ = 300 K, respectively. For the adiabatic boundary walls with gas molecules reflecting back diffusely, the components of the reflected velocity can be written as refs 37 and 38

19 $${U}_{re}=A{c}_{mw}$$

20 $${V}_{re}=B{c}_{mw}\,\cos \,\theta $$

21 $${W}_{re}=B{c}_{mw}\,\sin \,\theta $$

where $A=\sqrt{-\,\mathrm{ln}\,{R}_{f}}$, $B=\sqrt{-\,{\rm{l}}{\rm{n}}\,{R}_{f}}$, *θ* = 2*πR*_*f*_, ${c}_{wm}=\sqrt{2{\kappa }_{B}{T}_{re}/m}$, *R*_*f*_ is a random number, *m* is the mass of the molecules and *T*_*re*_ is the characteristic temperature of reflected surface which will be determined as follows.

Argon is the simple monatomic molecule gas with zero internal degree-of-freedom, so the energy of the molecules carrying can be given as

22 $$\begin{array}{cc}{E}_{in}=\sum _{i=1}^{n}{\varepsilon }_{in}^{i}, & {E}_{re}=\sum _{i=1}^{n}{\varepsilon }_{re}^{i}\end{array}$$

where *E*_*in*_ and *E*_*re*_ are the energy sums of the incident and reflected molecules, respectively. ${\varepsilon }_{in}^{i}$ and ${\varepsilon }_{re}^{i}$ are the incident and reflected translational energy of the molecule *i*, respectively.

23 $${\varepsilon }_{re}^{i}=0.5m({U}_{re}^{2}+{V}_{re}^{2}+{W}_{re}^{2})={\kappa }_{B}{T}_{re}({A}^{2}+{B}^{2})$$

where *κ*_*B*_ is the Boltzmann constant. For adiabatic boundary conditions with no energy exchange between molecules and the walls, we can obtain

24 $$\begin{array}{rcl}{E}_{in}={E}_{re} & = & 0.5\,\,m\sum _{i=1}^{n}({U}_{re}^{2}+{V}_{re}^{2}+{W}_{re}^{2})\\  & = & {\kappa }_{B}{T}_{re}\sum _{i=1}^{n}({A}^{2}+{B}^{2})\end{array}$$

Then, the characteristic temperature of reflected surface can be expressed as

25 $${T}_{re}={E}_{in}/[{\kappa }_{B}\sum _{i=1}^{n}({A}^{2}+{B}^{2})]$$

With the temperature obtained by Eq. (25), the complete diffuse reflection condition can be imposed on the walls of the nanolayers.
